# Systemic therapy in younger and elderly patients with advanced biliary cancer: sub-analysis of ABC-02 and twelve other prospective trials

**DOI:** 10.1186/s12885-017-3266-9

**Published:** 2017-04-12

**Authors:** Mairéad Geraldine McNamara, John Bridgewater, Andre Lopes, Harpreet Wasan, David Malka, Lars Henrik Jensen, Takuji Okusaka, Jennifer J. Knox, Dorothea Wagner, David Cunningham, Jenny Shannon, David Goldstein, Markus Moehler, Tanios Bekaii-Saab, Juan W. Valle

**Affiliations:** 1 Division of Molecular & Clinical Cancer Sciences, Institute of Cancer Sciences, The University of Manchester and The Christie NHS Foundation Trust, Manchester, M20 4BX UK; 2 UCL Cancer Institute, London, WCIE 6BT UK; 3 Cancer Research UK & UCL Cancer Trials Centre, London, WCIE 6BT UK; 4 Imperial Healthcare, London, WI2 ONN UK; 5 Institute Gustave Roussy, 94805 Villejuif, France; 6 Vejle Hospital, 7100 Vejle, Denmark; 7 National Cancer Center Hospital, Tokyo, 104-0045 Japan; 8 Princess Margaret Cancer Centre, Toronto, M5G 2M9 Canada; 9 Centre Hospitalier Universitaire Vaudois, CH-1011 Lausanne, Switzerland; 10 Royal Marsden Hospital, London, UK; 11 University of Sydney, Sydney, NSW 2006 Australia; 12 Prince of Wales Clinical School, University of New South Wales, Sydney, NSW 2052 Australia; 13 Universitätsmedizin Mainz, 55122 Mainz, Germany; 14 Mayo Clinic, Phoenix, AZ 85054 USA

**Keywords:** Biliary cancer, Younger patients, Elderly, Systemic therapy, Prospective trials

## Abstract

**Background:**

Outcomes in younger (<40 years) and elderly (≥70 years) patients with advanced biliary cancer (ABC) receiving palliative chemotherapy are unclear. This study assessed outcomes in those receiving monotherapy or combination therapy in thirteen prospective systemic-therapy trials.

**Methods:**

Multivariable analysis explored the impact of therapy on progression-free (PFS) and overall survival (OS) in two separate age cohort groups: <70 years and ≥70 years, and <40 years and ≥40 years.

**Results:**

Overall, 1163 patients were recruited (Jan 1997-Dec 2013). Median age of entire cohort: 63 years (range 23–85); 36 (3%) were <40, 260 (22%); ≥70. Combination therapy was platinum-based in nine studies. Among patients <40 and ≥70 years, 23 (64%) and 182 (70%) received combination therapy, respectively. Median follow-up was 42 months (95%-CI 37–51). Median PFS for patients <40 and ≥40 years was 3.5 and 5.9 months (*P* = 0.12), and OS was 10.8 and 9.7 months, respectively (*P* = 0.55). Median PFS for those <70 and ≥70 years was 6.0 and 5.0 months (*P* = 0.53), and OS was 10.2 and 8.8 months, respectively (*P* = 0.08). For the entire cohort, PFS and OS were significantly better in those receiving combination therapy: Hazard Ratio [HR]-0.66, 95%-CI 0.58–0.76, *P* < 0.0001 and HR-0.72, 95%-CI 0.63–0.82, *P* < 0.0001, respectively; and in patients ≥70 years: HR-0.54 (95%-CI 0.38–0.77, *P* = 0.001) and HR-0.60 (95%-CI 0.43–0.85, *P* = 0.004), respectively. There was no evidence of interaction between age and treatment for PFS (*P* = 0.58, *P* = 0.66) or OS (*P* = 0.18, *P* = 0.75).

**Conclusions:**

In ABC, younger patients are rare, and survival in elderly patients in receipt of systemic therapy for advanced disease, whether monotherapy or combination therapy, is similar to that of non-elderly patients, therefore age alone should not influence decisions regarding treatment.

## Background

Biliary tract cancers are rare and encompass cholangiocarcinoma, referring to cancers arising in the intrahepatic, perihilar, or distal biliary tree, gallbladder cancer and carcinoma of the ampulla of Vater [1]. Combination treatment with cisplatin/gemcitabine is currently considered standard of care for the treatment of patients with advanced biliary cancer (ABC), following the results of the randomised-controlled phase 3 trial, ABC-02, which reported a progression-free (PFS) and overall survival (OS) benefit for this combination over gemcitabine alone in 410 patients with ABC [2].

Comorbidities and age-related organ dysfunction are more often reported in elderly patients. There is often uncertainty regarding the benefits and risks of treatment in this subgroup [3, 4], which in turn may lead to a reluctance to implement chemotherapy, and particularly combination therapy, in these patients. Consequently they tend to represent a minority of those enrolled in clinical trials. There is thus less evidence to support treatment of elderly patients with cancer, who make up the majority of patients with this diagnosis.

For the more common cancers such as lung [5, 6] and colorectal cancer [7], there have been elderly-specific randomised-controlled trials and robust age-specific subgroup analyses of large studies, which provide guidance on treatment decisions in the clinical setting. However, there is a dearth of such data for rarer tumours such as ABC. In a progressively-ageing population, outcomes in elderly patients with ABC receiving palliative chemotherapy are unclear and can be a challenging therapeutic scenario for oncologists.

In addition, younger age may also influence outcomes in patients with a cancer diagnosis. In patients aged ≤30 years with breast cancer referred for surgery, for example, it has been reported that there is a greater chance of having an endocrine-unresponsive tumour and a significantly worse prognosis than those patients aged between 31 and 50 years [8]. It has also been described that younger patients (<40 years) with peripheral cholangiocarcinoma had a significantly worse survival rate than older patients who received surgical treatment [9]. However, information is lacking on the influence of age on the outcomes of younger patients receiving systemic therapy for ABC and so 40 years was chosen as age cut-off for further analysis based on publication by Yeh et al. [9].

The American Society of Clinical Oncology recently developed recommendations to improve evidence generation in older patients with cancer in response to a critical need identified by the Institute of Medicine [10]. Hence, the aim of this study was to assess outcomes (PFS and OS) of receipt of monotherapy versus combination therapy in younger (<40 years) and elderly patients (≥70 years) with ABC in ABC-02 and twelve other prospective trials of systemic therapy.

Seventy years was chosen as the age cut-off for elderly patients in this study due to the exponential rise in the prevalence of age-related changes between 70 and 75 years, and that approximately 90% of people demonstrate clinical signs of ageing by the age of 70 [11].

## Methods

Individual patient data from eleven international first-line clinical trials, and two using targeted therapies (with one study including eleven and another nine patients who received one prior line of therapy), in ABC (The International Biliary Tract Cancer Collaborators provided approval for the use of this data) were accessed for analysis (Table 1) [2, 12–23]. All trials were approved by appropriate research ethics committees and regulatory authorities and conducted in accordance with the Declaration of Helsinki. Baseline characteristics analysed included age, gender, Eastern Cooperative Oncology Group Performance Status (ECOG PS), disease stage (locally advanced versus metastatic), systemic therapy (monotherapy or combination). Site of primary (cholangiocarcinoma, gallbladder or ampulla of Vater), histology of tumour (adenocarcinoma versus other), previous therapy, haemoglobin, white blood cell, neutrophil count, and bilirubin were analysed and presented as part of a previous publication [24], where a model of neutrophils, disease stage, bilirubin, ECOG PS, haemoglobin, white blood cells, and gender were prognostic for PFS and OS, whereas age, site of primary, histology of tumour, previous therapy, and platelets were not [24]. Interrogation of those patients receiving combination cisplatin/gemcitabine has also been extensively analysed previously [24, 25]. The association between baseline categorical variables and age was tested using the Chi-squared test.

**Table 1:** Details of prospective trials included

Relevant publication	*N* ^a^	Age: Median (range)	Phase	Systemic Therapy
Bekaii-Saab et al. 2011 [12]	28	56 (26–79)	II, Non-randomised	Selumetinib
Goldstein et al. 2011 [13]	50	59 (39–78)	II, Non-randomised	Gemcitabine/Cisplatin
Jensen et al. 2012 [14]	46	66 (37–80)	II, Non-randomised	Gemcitabine/Oxaliplatin/Panitumumab/Capecitabine
Lassen et al. 2011 [15]	41	61 (35–75)	II, Non-randomised	Gemcitabine/Oxaliplatin/Capecitabine
Malka et al. 2014 (BINGO) [16]	150	62 (35–75)	II, Randomised	Gemcitabine/Oxaliplatin ± Cetuximab
Moehler et al. 2014 (AIO) [17]	102	64 (36–84)	II, Randomised	Gemcitabine ± Sorafenib
Okusaka et al. 2010 (BT22) [18]	83	66 (43–80)	II, Randomised	Gemcitabine ± Cisplatin
Peck et al. 2012 [19]	9	61 (31–83)	II, Non-randomised	Lapatinib
Rao et al. 2005 [20]	54	57 (36–76)	III, Randomised	5-Fluorouracil/Etoposide/Leucovorin versus Epirubicin/Cisplatin/5-Fluorouracil
Riechelmann et al. 2007 [21]	75	61 (37–84)	II, Non-randomised	Gemcitabine/Capecitabine
Ferraro et al. 2016 (TACTIC) [22]	48	64 (40–82)	II, Non-randomised	Gemcitabine/Cisplatin/Panitumumab
Valle et al. 2010 (ABC-02) [2]	410	63 (23–85)	III, Randomised	Gemcitabine ± Cisplatin
Wagner et al. 2009 [23]	72	62 (36–80)	II, Non-randomised	Gemcitabine/Oxaliplatin/5-Fluorouracil

^a^Due to non-availability of some data, 5 patients were not included in overall analysis

This study was an exploratory analysis based on available data. Detection of a specific effect size (hazard ratio) was not the target, and so power calculations were not used, as detection of an intended hazard ratio was not required. Progression-free survival [time from randomisation to progression or death, whichever happens first] and OS [time from randomisation to death] were analysed using Cox proportional hazards regression. Multivariable analysis was employed to explore the impact of age and therapy (monotherapy versus combination) on PFS and OS in four age cohorts; those <40 years (younger) versus ≥40 years and non-elderly (<70 years) versus elderly (≥70 years). The multivariable model was adjusted for the following variables: gender, ECOG PS, disease stage, haemoglobin, white blood cell count, neutrophil count, and bilirubin.

The Stata, version 14.1, statistical software package (Stata Corporation, College Station, Texas) was used to analyse the data.

## Results

### Patient characteristics

Overall, 1163 patients were recruited (January 1997–December 2013). Details on prospective studies included are contained within Table 1. Complete demographic data for individual trials is available within respective publications [2, 12–23].

The baseline patient characteristics for all patients are detailed in Table 2. The median age of the entire cohort was 63 years (range 23–85); 36 (3%) were <40, 260 (22%) were ≥70 and 18 (2%) were ≥80 years. Baseline characteristics/therapy received was balanced in all age cohorts (Table 2). Combination therapy was platinum-based in nine studies (*N* = 679 [58%]).

**Table 2:** Distribution of baseline characteristics by age group^a^

	Covariate	<40 years Total *N* = 36 *N* (%)	≥40 years Total *N* = 1127 *N* (%)	*P*-value^b^	<70 years Total *N* = 903 *N* (%)	≥70 years Total *N* = 260 *N* (%)	*P*-value^b^
Gender	Female	16 (44)	597 (53)	0.31	482 (53)	131 (50)	0.39
	Male	20 (56)	530 (47)		421 (47)	129 (50)	
ECOG performance status	0	10 (28)	350 (31)	0.59	285 (32)	75 (29)	0.24
	1	16 (44)	572 (51)		455 (50)	133 (51)	
	2	4 (11)	81 (7)		60 (7)	25 (10)	
	Not available	6 (17)	124 (11)		103 (11)	27 (10)	
Disease Stage	Locally advanced	9 (25)	295 (26)	0.80	233 (26)	71 (27)	0.43
	Metastatic	27 (75)	800 (71)		652 (72)	175 (67)	
	Not available	0 (0)	32 (3)		18 (2)	14 (5)	
Treatment	Combination	23 (64)	809 (72)	0.20	650 (72)	182 (70)	0.48
	Monotherapy	12 (33)	301 (27)		239 (26)	74 (28)	
	Not available	1 (3)	17 (2)		14 (2)	4 (2)	

ECOG performance status: Eastern Cooperative Oncology Group Performance Status

^a^Due to rounding, all percentages in Table 2 may not equal 100%. ^b^Chi-squared test; performed excluding the category “not available”

The median follow-up time for all patients was 42 months (95%-Confidence Interval [CI] 37–51).

### Progression-free and Overall Survival

The median PFS for the entire cohort [*N* = 1163] was 5.8 months (95%-CI 5.5–6.2).

The median PFS for patients aged <40 and ≥40 years was 3.5 (95%-CI 2.9–5.6) and 5.9 months (95%-CI 5.5–6.4) (*P* = 0.12) and for those <70 and ≥70 years, 6.0 (95%-CI 5.5–6.4) and 5.0 months (95%-CI 4.2–6.4), respectively (*P* = 0.53).

The 6-month PFS rate was 26% (95%-CI 13–41) and 49% (95%-CI 46–52) in the <40 and ≥40 year old cohort, respectively. The 6-month PFS rate was 50% (46–5253) and 45% (95%-CI 39–51) in the <70 and ≥70 year old cohort, respectively.

**Fig. 1:**
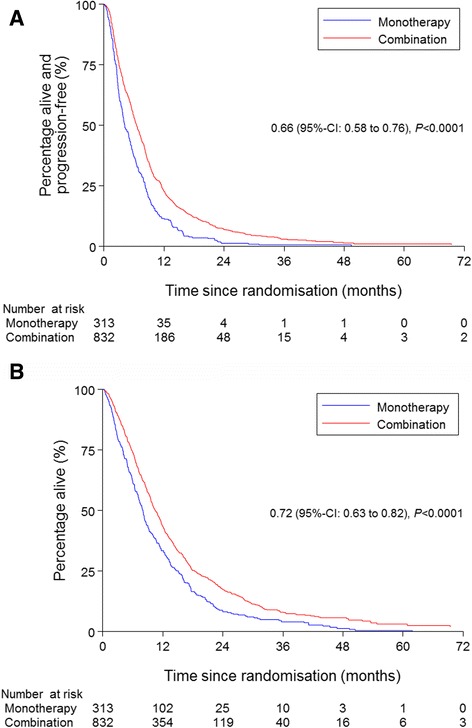
Kaplan-Meier estimates of progression-free survival (**a**) and overall survival (**b**) in patients with advanced biliary tract cancer who received monotherapy versus combination therapy

The median PFS in the entire cohort for those receiving monotherapy and combination therapy was 4.2 (95%-CI 3.7–5.1) and 6.5 months (95%-CI 6.0–7.1), respectively (*P* < 0.0001) (Fig. 1a).

The median OS for the entire cohort was 9.8 months (95%-CI 9.2–10.5).

The median OS for patients <40 and ≥40 years was 10.8 (95%-CI 5.4–12.7) and 9.7 months (95%-CI 9.2–10.4) (*P* = 0.55) and for patients <70 and ≥70 years, 10.2 (95%-CI 9.6–11.1) and 8.8 months (95%-CI 7.9–9.6), respectively (*P* = 0.08).

The 6-month OS rate was 60% (95%-CI 42–74) and 71% (95%-CI 68–74) in the <40 and ≥40 year old cohort respectively. The 6-month OS rate was 72% (68–74) and 68% (95%-CI 61–73) in the <70 and ≥70 year old cohort, respectively.

The median OS in the entire cohort for those receiving monotherapy and combination therapy was 8.1 (95%-CI 7.1–8.7) and 10.6 months (95%-CI 9.8–11.4), respectively (*P* < 0.0001) (Fig. 1b).

**Fig. 2:**
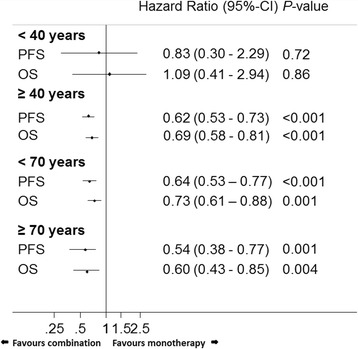
Hazard ratios (combination versus monotherapy) for progression-free and overall survival in different age cohorts. (Adjusted for gender, Eastern Co-operative Oncology Group performance status, disease stage, haemoglobin, white blood cell count, neutrophil count, and bilirubin). CI: confidence interval, PFS: progression-free survival, OS: overall survival

In the entire population, the PFS and OS were significantly better in those patients receiving combination therapy in the individual age groups; ≥40, <70 and ≥70 years, but not in those aged <40 years (Fig. 2).

Similarly, in a sub-analysis of those patients receiving the cisplatin/gemcitabine combination (*N* = 297) versus those receiving gemcitabine alone (*N* = 258), the PFS and OS were significantly better in those patients receiving combination therapy in the individual age groups; ≥40 (both *P* < 0.001), <70 (*P* < 0.001 and *P* = 0.002 respectively) and ≥70 years (*P* = 0.003 and *P* = 0.014 respectively), but not in those aged <40 years (*P* = 0.71 and *P* = 0.72 respectively).

### Prognostic factors

Age was not prognostic for PFS or OS in those receiving monotherapy (*P* = 0.49 and *P* = 0.08 respectively) or combination therapy (*P* = 0.67 and *P* = 0.27 respectively), and there was no evidence of interaction between age and treatment (monotherapy and combination therapy) in the <40 and ≥40 years age groups for PFS (*P* = 0.58) or OS (*P* = 0.18) or in the <70 and ≥70 years age groups for PFS (*P* = 0.66) or OS (*P* = 0.75). There was no evidence of an interaction between tumour location and age on PFS (Interaction with age model *P* value: Hilar: *P* = 0.46, Gallbladder: *P* = 0.33, Extrahepatic: *P* = 0.49, Ampulla of Vater: *P* = 0.20) or OS (Hilar: *P* = 0.53, Gallbladder: *P* = 0.99, Extrahepatic: *P* = 0.60, Ampulla of Vater: *P* = 0.56).

In the overall population, on multivariable analysis, PFS was worse in those patients with metastatic disease versus those with locally advanced disease in those receiving monotherapy (Hazard Ratio [HR] 1.35, 95%-CI 0.99–1.83, *P* = 0.06) and combination therapy (HR 1.42, 95%-CI 1.16–1.75, *P* = 0.001). Overall survival was also worse in those patients with metastatic disease versus those with locally advanced disease in those receiving monotherapy (HR 1.54, 95%-CI 1.12–2.12, *P* = 0.01) and combination therapy (HR 1.41, 95%-CI 1.14–1.74, *P* = 0.001).

In patients <40 years, ECOG PS was prognostic for PFS and OS and in patients ≥40 years, stage was prognostic for PFS, and stage and ECOG PS were prognostic for OS (Table 3).

**Table 3:** Multivariable analysis for progression-free and overall survival (<40 and ≥40 years)^a^

	Covariate	PFS <40 years	PFS ≥40 years	OS <40 years	OS ≥40 years
Gender (reference; female)	Female vs Male	HR 0.41 (95% CI 0.13–1.33, *P* = 0.14)	HR 1.02 (95% CI 0.88–1.18, *P* = 0.81)	HR 1.11 (95% CI 0.38–3.20, *P* = 0.85)	HR 1.14 (95% CI 0.97–1.32, *P* = 0.10)
ECOG performance status (reference; 0)	0 vs 1	HR 4.94 (95% CI 1.13–21.52)	HR 1.05 (95% CI 0.89–1.23)	HR 3.59 (95% CI 0.79–16.37)	HR 1.02 (95% CI 0.86–1.20)
	0 vs 2	HR 15.89 (95% CI 2.19–115.33) [*P* = 0.01]	HR 1.39 (95% CI 1.04–1.85) [*P* = 0.10]	HR 113.11 (95% CI 7.99–1600.53) [*P* = 0.001]	HR 1.87 (95% CI 1.40–2.50) [*P* = 0.0002]
Disease stage (reference; locally advanced)	Locally advanced vs Metastatic	HR 3.17 (95% CI 0.73–13.77, *P* = 0.12)	HR 1.38 (95% CI 1.17–1.64, *P* < 0.001)	HR 4.91 (95% CI 0.77–31.07, *P* = 0.09)	HR 1.40 (95% CI 1.18–1.68, *P* < 0.001)

*PFS* Progression-free survival, *OS* Overall survival, *ECOG PS* Eastern Cooperative Oncology Group Performance Status

^a^The multivariable model was adjusted for the following variables; treatment, haemoglobin, white blood cell count, neutrophil count, and bilirubin

In patients <70 years, stage and ECOG PS were prognostic for PFS and OS; and in patients ≥70 years, they were prognostic for OS (Table 4).

**Table 4:** Multivariable analysis for progression-free and overall survival (<70 and ≥70 years)^a^

	Covariate	PFS <70 years	PFS ≥70 years	OS <70 years	OS ≥70 years
Gender (reference; female)	Female vs Male	HR 1.00 (95% CI 0.84–1.18, *P* = 0.98)	HR 1.14 (95% CI 0.83–1.56, *P* = 0.42)	HR 1.12 (95% CI 0.94–1.33, *P* = 0.22)	HR 1.33 (95% CI 0.97–1.84, *P* = 0.08)
ECOG performance status (reference; 0)	0 vs 1	HR 1.10 (95% CI 0.92–1.32)	HR 0.86 (95% CI 0.60–1.24)	HR 1.08 (95% CI 0.90–1.31)	HR 0.81 (95% CI 0.57–1.16)
	0 vs 2	HR 1.58 (95% CI 1.13–2.20) [*P* = 0.04]	HR 1.03 (95% CI 0.58–1.83) [*P* = 0.65]	HR 2.02 (95% CI 1.45–2.82) [*P* = 0.001]	HR 1.84 (95% CI 1.03–3.28) [*P* = 0.02]
Disease stage (reference; locally advanced)	Locally advanced vs Metastatic	HR 1.46 (95% CI 1.20–1.78, *P* < 0.001)	HR 1.21 (95% CI 0.86–1.69, *P* = 0.28)	HR 1.48 (95% CI 1.20–1.82, *P* < 0.001)	HR 1.49 (95% CI 1.05–2.12, *P* = 0.03)

*PFS* rogression-free survival, *OS*O verall survival, *ECOG PS* Eastern Cooperative Oncology Group Performance Status

^a^The multivariable model was adjusted for the following variables; treatment, haemoglobin, white blood cell count, neutrophil count, and bilirubin

## Discussion

Information is lacking on outcomes of patients with a diagnosis of ABC who are <40 years receiving palliative chemotherapy, and as the global population ages, there is also an increasing focus on the need to evaluate treatment outcomes in older patients with cancer. No prospective studies report on the efficacy and safety of palliative chemotherapy in younger patients with ABC and only a few studies; none prospective, have reported on efficacy and safety of palliative chemotherapy in elderly patients with ABC [26–28]. Although gemcitabine-platinum doublet therapy is now the most common standard therapeutic option for patients with ABC [2, 18], there may be resistance among physicians in general clinical practice to prescribe combination rather than monotherapy in an older population due to perceptions of potential increased toxicity and increased presence of comorbidities.

Age was not prognostic for PFS or OS in those receiving monotherapy or combination therapy. Progression-free and OS in patients receiving combination versus monotherapy were statistically significantly better in those ≥40 years and in those <70 and ≥70 years in the entire population and in a sub-group analysis of those receiving the cisplatin/gemcitabine combination versus gemcitabine alone. The small sample size in those patients <40 years precluded a significant outcome and may be associated with a relevant bias and so results may be of limited value in this subgroup. Similar percentages of patients with locally advanced and metastatic disease were included within the <40 and ≥40 year subgroups, and so this would not account for results obtained. No family history was reported in patient subgroups, but the life-time risk of bile duct cancers in patients with Lynch syndrome is only approximately 2% [29], and should not be relevant here. Given that this was an international collaboration, this does highlight the rarity of ABC in this age group, at least in those included in these prospective clinical trials for ABC [3%] [2, 12–23].

There were only eighteen patients aged ≥80 years, therefore meaningful subgroup analysis was not possible and the benefit of combination versus monotherapy remains unclear in this age cohort. Eastern Cooperative Oncology Group PS was prognostic for OS in all of the four age cohorts and the presence of metastatic rather than locally advanced disease had an adverse prognostic effect on OS in those ≥40, <70 and ≥70 years, which is similar to findings from ABC-02 [2].

Limitations of this study are lack of toxicity and comorbidity analysis, and consequently the cost of these toxicities and potential inpatient stays, to the elderly population, cannot be estimated. However, toxicity data and treatment duration have been published previously within individual manuscripts [2, 12–23], and it is unlikely that patients with significant comorbidities were included in these prospective studies due to clinical trial eligibility criteria. Of course, selection bias may then be inherent in prospective studies, but it has recently been reported in a large retrospective study that active therapy, when given, in older patients with ABC, is associated with similar survival benefits, irrespective of age [28]. It has also recently been reported that the survival advantage of cisplatin/gemcitabine compared to gemcitabine alone was not associated with an improvement or deterioration of quality of life in ABC-02 [30].

Another limiting factor of this analysis was the heterogeneity of the treatment given in the included series, but the OS data reported for monotherapy and combination therapy in patients with ABC is not dissimilar within this study to that reported in ABC-02, and the addition of chemotherapy or targeted therapy to the established ABC-02 regimen, or others, has not lead to significant improvements in survival to date [2, 12–23, 31]. The shorter PFS reported in the current study in patients receiving monotherapy and combination therapy may be attributable to scanning interval variation. In ABC-02, this was 12 weekly [2], whereas in Okusaka et al. [18], imaging was performed every 6 weeks [18, 31].

Data on therapy given following completion of respective therapies is not available, but as OS in the different age cohorts was comparable, it is likely that patients included in these clinical trials were treated similarly on progression. However, given the rarity of this diagnosis, this study was a significant effort to address the role of systemic therapy in those <40 and ≥70 years in thirteen prospective trials, five of which were randomised.

## Conclusions

In patients with ABC, cautious interpretation of data is required in relation to monotherapy versus combination therapy in those patients <40 years, due to the limited number of patients in this subgroup, and more study in this age cohort is necessary. Other age-related co-variables such as primary sclerosing cholangitis and the potential presence of breast cancer susceptibility genes 1/2 (*BRCA1/2)* mutations may be enriched in those <40 years and may confound OS outcomes. Survival in elderly patients (≥70 years) in receipt of systemic therapy for ABC is similar to that of non-elderly patients (<70 years), including significant benefit from combination therapies over monotherapy in the age strata ≥70 years similar to the overall population. Therefore, age alone should not dictate decisions on treatment, and thus elderly patient participation in clinical trials for ABC is appropriate, acknowledging that this study provides data on a clinical trial eligible population ≥ 70 years (e.g. very fit). Comprehensive geriatric assessment tools [32–34], incorporating an understanding of older patient’s individual health profiles, their practical/social needs, and their wishes, rather than just their chronological age, need to be an integral component of the complicated decision-making processes when deciphering which patients may benefit from potentially more toxic combination therapy, and should form a useful adjunct to future elderly patient-focused therapeutic trials with ABC. The utilisation of assessment tools to better predict tolerance and toxicity to chemotherapy should also be considered [35–37].

## Abbreviations

ABC: Advanced biliary cancer
CI: Confidence interval
ECOG PS: Eastern Cooperative Oncology Group Performance Status
HR: Hazard ratio
OS: Overall survival
PFS: Progression-free survival

## References

[1] Siegel R, Naishadham D, Jemal A (2013). Cancer statistics, 2013. CA Cancer J Clin.

[2] Valle J, Wasan H, Palmer DH, Cunningham D, Anthoney A, Maraveyas A, Madhusudan S, Iveson T, Hughes S, Pereira SP (2010). Cisplatin plus Gemcitabine versus Gemcitabine for biliary tract cancer. N Engl J Med.

[3] Sehl M, Sawhney R, Naeim A (2005). Physiologic aspects of aging: impact on cancer management and decision making, part II. Cancer J.

[4] Sawhney R, Sehl M, Naeim A (2005). Physiologic aspects of aging: impact on cancer management and decision making, part I. Cancer J.

[5] Camerinin A, Puccetti C, Donati S, Valsuani C, Petrella MC, Tartarelli G, Puccinelli P, Amoroso D (2015). Metronomic oral vinorelbine as first-line treatment in elderly patients with advanced non-small cell lung cancer: results of a phase II trial (MOVE trial). BMC Cancer.

[6] Cuffe S, Booth CM, Peng Y, Darling GE, Li G, Kong W, Mackillop WJ, Shepherd FA (2012). Adjuvant chemotherapy for non-small-cell lung cancer in the elderly: a population-based study in Ontario. Canada J Clin Oncol.

[7] Cunningham D, Lang I, Marcuello E, Lorusso V, Ocvirk J, Shin DB, Jonker D, Osborne S, Andre N, Waterkamp D (2013). Bevacizumab plus capecitabine versus capecitabine alone in elderly patients with previously untreated metastatic colorectal cancer (AVEX): an open-label, randomised phase 3 trial. Lancet Oncol.

[8] Yao Y, Cao M, Fang H, Xie J (2015). Breast Cancer in 30-year-old or younger patients: clinicopathologic characteristics and prognosis. World J Surg Oncol.

[9] Yeh CN, Jan YY, Chen MF (2004). Influence of age on surgical treatment of peripheral cholangiocarcinoma. Am J Surg.

[10] Hurria A, Levit LA, Dale W, Mohile SG, Muss HB, Fehrenbacher L, Magnuson A, Lichtman SM, Bruinooge SS, Soto-Perez-de-Celis E (2015). Improving the evidence base for treating older adults with cancer: American Society of Clinical Oncology statement. J Clin Oncol.

[11] Balducci L, Extermann M (2000). Management of cancer in the older person: a practical approach. Oncologist.

[12] Bekaii-Saab T, Phelps MA, Li X, Saji M, Goff L, Kauh JS, O'Neil BH, Balsom S, Balint C, Liersemann R (2011). Multi-Institutional Phase II Study of Selumetinib in Patients With Metastatic Biliary Cancers. J Clin Oncol.

[13] Goldstein D, Gainford MC, Brown C, Tebbutt N, Ackland SP, van Hazel G, Jefford M, Abdi E, Selva-Nayagam S, Gebski V (2011). Fixed-dose-rate gemcitabine combined with cisplatin in patients with inoperable biliary tract carcinomas. Cancer Chemother Pharmacol.

[14] Jensen LH, Lindebjerg J, Ploen J, Hansen TF, Jakobsen A (2012). Phase II marker-driven trial of panitumumab and chemotherapy in KRAS wild-type biliary tract cancer. Ann Oncol.

[15] Lassen U, Jensen LH, Sorensen M, Rohrberg KS, Ujmajuridze Z, Jakobsen A (2011). A phase I-II dose escalation study of fixed-dose rate gemcitabine, oxaliplatin and capecitabine every two weeks in advanced cholangiocarcinomas. Acta Oncol.

[16] Malka D, Cervera P, Foulon S, Trarbach T, de la Fouchardière C, Boucher E, Fartoux L, Faivre S, Blanc JF, Viret F (2014). Gemcitabine and oxaliplatin with or without cetuximab in advanced biliary-tract cancer (BINGO): a randomised, open-label, non-comparative phase 2 trial. The Lancet Oncology.

[17] Moehler M, Maderer A, Schimanski C, Kanzler S, Denzer U, Kolligs FT, Ebert MP, Distelrath A, Geissler M, Trojan J (2014). Gemcitabine plus sorafenib versus gemcitabine alone in advanced biliary tract cancer: A double-blind placebo-controlled multicentre phase II AIO study with biomarker and serum programme. Eur J Cancer.

[18] Okusaka T, Nakachi K, Fukutomi A, Mizuno N, Ohkawa S, Funakoshi A, Nagino M, Kondo S, Nagaoka S, Funai J (2010). Gemcitabine alone or in combination with Cisplatin in patients with biliary tract cancer: a comparative multicentre study in Japan. Br J Cancer.

[19] Peck J, Wei L, Zalupski M, O'Neil B, Villalona Calero M, Bekaii-Saab T (2012). HER2/neu may not be an interesting target in biliary cancers: results of an early phase II study with lapatinib. Oncology.

[20] Rao S, Cunningham D, Hawkins RE, Hill ME, Smith D, Daniel F, Ross PJ, Oates J, Norman AR (2005). Phase III study of 5FU, etoposide and leucovorin (FELV) compared to epirubicin, cisplatin and 5FU (ECF) in previously untreated patients with advanced biliary cancer. Br J Cancer.

[21] Riechelmann RP, Townsley CA, Chin SN, Pond GR, Knox JJ (2007). Expanded phase II trial of gemcitabine and capecitabine for advanced biliary cancer. Cancer.

[22] Ferraro D, Goldstein D, O’Connell RL, Hill ME, Smith D, Daniel F, Ross PJ, Oates J, Norman AR (2016). TACTIC: a multicentre, open-label, single arm phase II trial of panitumumab, cisplatin, and gemcitabine in biliary tract cancer. Cancer Chemother Pharmacol.

[23] Wagner AD, Buechner-Steudel P, Moehler M, Schmalenberg H, Behrens R, Fahlke J, Wein A, Behl S, Kuss O, Kleber G (2009). Gemcitabine, oxaliplatin and 5-FU in advanced bile duct and gallbladder carcinoma: two parallel, multicentre phase-II trials. Br J Cancer.

[24] Bridgewater J, Lopes A, Wasan H, Malka D, Jensen L, Okusaka T, Knox J, Wagner D, Cunningham D, Shannon J (2016). Prognostic factors for progression-free and overall survival in advanced biliary tract cancer. Ann Oncol.

[25] Valle JW, Furuse J, Jitlal M, Beare S, Mizuno N, Wasan H, Bridgewater J, Okusaka T (2014). Cisplatin and gemcitabine for advanced biliary tract cancer: a meta-analysis of two randomised trials. Ann Oncol.

[26] Kuriyama H, Kawana K, Taniguchi R, Jono F, Sakai E, Ohubo H, Suzuki H, Kobayashi S, Murata Y, Inamori M (2011). Single-agent gemcitabine in elderly patients with unresectable biliary tract cancer. Hepato-Gastroenterology.

[27] Kou T, Kanai M, Ikezawa K, Ajiki T, Tsukamoto T, Toyokawa H, Yazumi S, Terajima H, Furuyama H, Nagano H (2014). Comparative outcomes of elderly and non-elderly patients receiving first-line palliative chemotherapy for advanced biliary tract cancer. J Gastroenterol Hepatol.

[28] Horgan A, Knox J, Aneja P, Le L, McKeever E, McNamara M (2015). Patterns of care and treatment outcomes in older patients with biliary tract cancer. Oncotarget.

[29] Umar A, Boland CR, Terdiman JP, Syngal S, de la Chapelle A, Rüschoff J, Fishel R, Lindor NM, Burgart LJ, Hamelin R (2004). Revised Bethesda guidelines for hereditary nonpolyposis colorectal cancer (Lynch syndrome) and microsatellite instability. J Natl Cancer Inst.

[30] Bridgewater J, Lopes A, Palmer D, Cunningham D, Anthoney A, Maraveyas A, Madhusudan S, Iveson T, Valle J, Wasan H (2016). Quality of life, long-term survivors and long-term outcome from the ABC-02 study. Br J Cancer.

[31] Furuse J, Okusaka T, Bridgewater J, Taketsuna M, Wasan H, Koshiji M, Valle J (2011). Lessons from the comparison of two randomized clinical trials using gemcitabine and cisplatin for advanced biliary tract cancer. Crit Rev Oncol Hematol..

[32] Chaibi P, Magne N, Breton S, Chebib A, Watson S, Duron JJ, Hannoun L, Lefranc JP, Piette F, Menegaux F (2011). Influence of geriatric consultation with comprehensive geriatric assessment on final therapeutic decision in elderly cancer patients. Crit Rev Oncol Hematol.

[33] Extermann M, Hurria A (2007). Comprehensive geriatric assessment for older patients with cancer. J Clin Oncol.

[34] Kenis C, Bron D, Libert Y, Decoster L, Van Puyvelde K, Scalliet P, Cornette P, Pepersack T, Luce S, Langenaeken C (2013). Relevance of a systematic geriatric screening and assessment in older patients with cancer: results of a prospective multicentric study. Ann Oncol.

[35] Hurria A, Togawa K, Mohile SG, Owusu C, Klepin HD, Gross CP, Lichtman SM, Gajra A, Bhatia S, Katheria V (2011). Predicting chemotherapy toxicity in older adults with cancer: a prospective multicentre study. J Clin Oncol.

[36] Hurria A, Mohile S, Gajra A, Klepin H, Muss H, Chapman A, Feng T, Smith D, Sun CL, De Glas N (2016). Validation of a prediction tool for chemotherapy toxicity in older adults with cancer. J Clin Oncol.

[37] Extermann M, Boler I, Reich RR, Lyman GH, Brown RH, DeFelice J, Levine RM, Lubiner ET, Reyes P, Schreiber FJ (2012). Predicting the risk of chemotherapy toxicity in older patients: the Chemotherapy Risk Assessment Scale for High-Age Patients (CRASH) score. Cancer.

